# Rectification of Images Distorted by Microlens Array Errors in Plenoptic Cameras

**DOI:** 10.3390/s18072019

**Published:** 2018-06-23

**Authors:** Suning Li, Yanlong Zhu, Chuanxin Zhang, Yuan Yuan, Heping Tan

**Affiliations:** 1 School of Energy Science and Engineering, Harbin Institute of Technology, 92 West Dazhi Street, Harbin 150001, China; 16b902036@stu.hit.edu.cn (S.L.); 17S002073@stu.hit.edu.cn (Y.Z.); chuanxin@hit.edu.cn (C.Z.); tanheping@hit.edu.cn (H.T.); 2 Key Laboratory of Aerospace Thermophysics, Ministry of Industry and Information Technology, Harbin Institute of Technology, 92 West Dazhi Street, Harbin 150001, China

**Keywords:** plenoptic camera, microlens array, distortion rectification, calibration, light-field image processing

## Abstract

A plenoptic cameras is a sensor that records the 4D light-field distribution of target scenes. The surface errors of a microlens array (MLA) can cause the degradation and distortion of the raw image captured by a plenoptic camera, resulting in the confusion or loss of light-field information. To address this issue, we propose a method for the local rectification of distorted images using white light-field images. The method consists of microlens center calibration, geometric rectification, and grayscale rectification. The scope of its application to different sized errors and the rectification accuracy of three basic surface errors, including the overall accuracy and the local accuracy, are analyzed through simulation of imaging experiments. The rectified images have a significant improvement in quality, demonstrating the provision of precise light-field data for reconstruction of real objects.

## 1. Introduction

Plenoptic cameras based on microlens arrays (MLAs), which apply light-field imaging technology, can acquire and display multi-angle light radiation intensity distributions from spatial targets in a single exposure [1,2]. In contrast to conventional camera sensors that only sample spatial information, they can simultaneously record the 4D light-field including 2D spatial information and 2D angular information. The retention of angular information of light radiation provides the necessary light-field data for multi-view imaging, digital refocusing, and 3D reconstruction of a target scene [2,3,4]. In a plenoptic camera, a MLA is added between the main lens and the image sensor to capture the raw image recording the complete light-field, which is composed of a series of sub-images formed by the respective microlenses arranged in a specific sequence. The positions of the sub-images correspond to the spatial light-field information, and the positions of the pixels beneath the sub-images correspond to the angular light-field information. Owing to this correlation, the MLA intrinsic and extrinsic parameters determine image quality and subsequent light-field decoding and reconstruction results, so it is important that the MLA itself has a high surface precision and maintains strict azimuth alignment.

In recent years, numerous reconstruction algorithms of complex scenes have been developed using light-field image data [5,6,7]. Plenoptic cameras are gradually being applied in engineering areas such as high temperature flame measurement [8,9], target recognition [10], particle image velocimetry [11,12], and real-time monitoring [13,14]. In order to effectively utilize the light-field data in the image processing and reconstruction process, the raw image should accurately record the light-field information of the measured target. However, due to manufacturing and assembly defects in the MLAs [15,16,17], different distortions appear in the actual images acquired using them, including blurring and aliasing caused by the orientation misalignments between the MLA and the image sensor [18], as well as changes in brightness, resolution and spot position owing to microlens surface errors [19,20]. The degradation and distortion of the raw image makes a large amount of data information confused or missing, leading to a significant reduction in the reconstruction accuracy and efficiency of the target flow field [21,22,23]. Therefore, it is necessary to rectify the distorted light-field image caused by the MLA errors. Differences in 2D image correction method [24,25,26], the rectification of light-field images, requires special attention to the distortion of the microlens sub-image. Jin et al. [27] presented a correction method for the raw image based on the commercial plenoptic camera Lytro. By estimating the rotation error angle of the MLA relative to the image sensor plane, the light-field image was integrally rotated to align with the pixel axis, eliminating the image distortion. Dansereau et al. [28] established a lens distortion model of the plenoptic camera and concluded that the light-field image was primarily affected by directionally dependent radial distortion. They further proposed a method for rectifying the ray projection errors of the decoded image. Cho et al. [29] described a method to calibrate the microlens images in a hexagonal arrangement, which can achieve sub-pixel precision locating of the microlens centers. The experimental results showed that the microlens centers were non-uniform, and the author inferred that this could be attributed to slight shape difference between the microlenses caused by manufacturing defects. Li et al. [30] analyzed the system imaging characteristics under MLA assembly error conditions, and provided proper quality evaluation indexes and correction functions for the error in the light-field image.

It is known from the aforementioned studies that current rectification methods generally idealize the MLA surface parameters and consider only the image distortion from the azimuth deviation between the integral MLA and sensor plane, while neglecting the microlens surface error. In fact, the MLA surface error and its image distortion are not consistent. On the one hand, owing to various machining techniques and materials, and the complicated structures, there are local differences in the MLA surface errors that result in different error forms and magnitudes for each microlens [15,31]. On the other hand, the same error on different microlenses deteriorates the corresponding sub-images differently. The trend of change in light-field image quality is related to the error type, size and spatial direction [20]. Therefore, integral methods cannot effectively rectify the local degradation caused by microlens surface errors, and moreover, such approaches generate problems of large computational complexity, insignificant correction effects, or excessive correction.

In order to reverse the effects of the MLA surface error and restore the light-field data of the space target, in this paper, we propose a new rectification method of distorted image for the MLA errors. The rectification method, which directly utilizes the raw white image, extracts coordinate and grayscale information of the feature points and ideal reference points for each sub-image. With the relevant information, the error sub-image geometric rectification and grayscale rectification matrices are determined, and thus the geometry and grayscale deviation of the light-field image can be locally rectified. Further, based on a ray-tracing simulation imaging system [32], we develop a MLA surface error model to analyze the rectification accuracy and its applicable error range under different error conditions. The proposed method is verified by comparing the real object images before and after rectification.

## 2. Models

### 2.1. Light-Field Imaging Model

**Figure 1:**
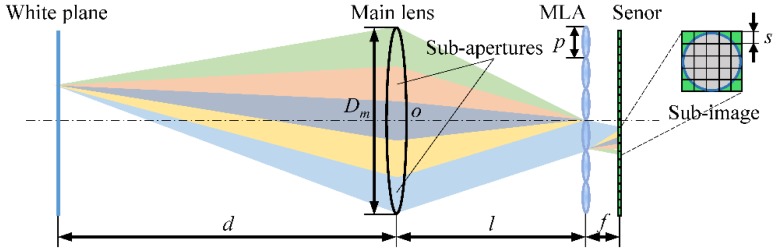
Physical model of plenoptic camera imaging system.

In this paper, on the basis of the Plenoptic Camera 1.0 designed by Ng et al. [2], we establish a physical model of plenoptic camera imaging system, which is used for light-field imaging simulation in the calibration and rectification process. As depicted in Figure 1, the imaging model mainly includes a main lens, a MLA and an image sensor, where the MLA is placed at the imaging plane of the main lens and the image sensor is coupled at one focal length behind the MLA. The MLA divides the main lens pupil into several sub-apertures. Each microlens samples the rays from multiple directions at a certain point through every sub-aperture, and finally, projects these rays onto the image sensor to form a sub-image, as shown in Figure 1. To obtain the raw white image required to rectify distorted light-field images, a white plate is placed at the distance of *d* = 2.5 m from the main lens, with the center on the system optical axis and the corresponding image distance of *l* = 109.6 mm. The plate size is 40 × 40 cm, the surface is diffuse reflective, and the reflectivity is 1.0. The other related parameters of the model may be found by referring to a previous report [20].

### 2.2. MLA Surface Error Model

**Figure 2:**
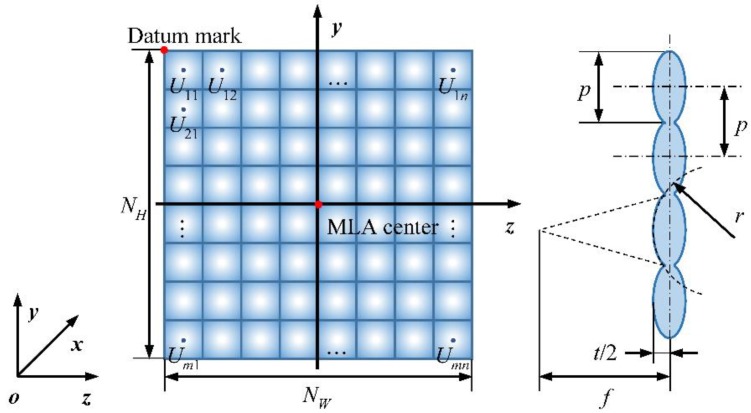
MLA design model and its coordinate system.

Figure 2 demonstrates the design model of the MLA used in this paper. The entire MLA consists of *N_W_* × *N_H_* square-aperture microlenses in a matrix arrangement. The length and center pitch of the microlenses are both *p*. The two sides of the microlenses are spherical surfaces with radius-of-curvature *r*, the microlens thickness is *t*, and the focal length is *f*. The main parameters of the MLA model are listed in Table 1. Each unit of the MLA is labelled as *U_m_*_,*n*_, which indicates that the unit is located in the *m*-th row and *n*-th column of the MLA, where *m* = 1, 2, …, *N_H_* and *n* = 1, 2, …, *N_W_*. We establish the MLA coordinate system *o*-*xyz* with the MLA center as the origin *o*. The *x*-axis passes through the optical axis of the main lens, and the *y*- and *z*-axes are parallel to the square grids of the microlenses, as shown in Figure 2. Setting the point $\left(0,y_{0},z_{0}\right)$ in the upper left corner of the MLA as the datum mark, the center coordinates of the microlens *U_m_*_,*n*_ are $\left(0,y_{0}-(m-1/2)p,z_{0}+(n-1/2)p\right)$, and its surface can be described by a standard spherical surface equation:

(1) $${\left[x\mp \left(r-t/2\right)\right]}^{2}+{\left[y-\left(y_{0}-(m-1/2)p\right)\right]}^{2}+{\left[z-\left(z_{0}+(n-1/2)p\right)\right]}^{2}=r^{2}$$

where the coordinates $y_{0}$ and $z_{0}$ can be calculated from $y_{0}=N_{H}p/2$ and $z_{0}=-N_{W}p/2$, respectively. Equation (1) with a negative sign for the term (r−t/2) signifies the microlens incident-light surface, whereas the equation with a positive sign signifies the transmitted-light surface.

However, due to manufacturing deviations in the production process, the MLA surface parameters do not perfectly coincide with the design values, resulting in different local surface errors, such as coordinate distortion [15], curvature variation [16], centroid shift [33,34,35], or irregular deformation [36]. We develop three basic error models that contain pitch error, radius-of-curvature error and decenter error, according to the arrangement and geometric features of the MLA used in this paper. The actual surface error can be characterized with a combination of these basic errors.

Under the condition that the other parameters of the MLA are constant, if the pitch between the adjacent microlenses changes, it is called the pitch error, Δ*p*; if the radius-of-curvature of the microlens deviates from the standard value, it is called the radius-of-curvature error, Δ*r*; if a certain offset occurs between the spherical centers on both sides of the microlens, it is called the decenter error, *δ*. Table 2 shows the mathematical description of the error models, where the negative sign of the term (*r* − *t*/2) indicates the incident-light surface equation of the error microlens *U_m_*_,*n*_, and the positive sign indicates the transmitted-light surface equation; *α* and *β* are the angles between the pitch error Δ*p* and the decenter error *δ* and the horizontal direction, respectively. As seen in Table 2, the pitch error and decenter error change the center position of the microlens, which causes the microlens optical axis to shift or tilt. The radius-of-curvature error only affects the spherical radius of the microlens, and consequently alters its focal length.

## 3. Rectification Method

**Figure 3:**
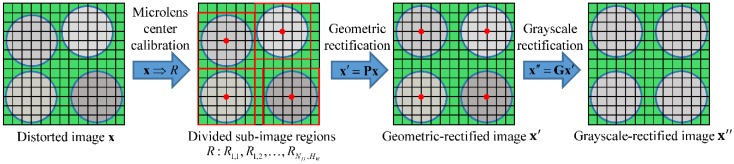
Schematic of the rectification procedure for a distorted light-field image.

In the previous studies, we analyzed the local imaging characteristics and degradation mechanism of the MLA surface error. The results showed that the error caused major distortions such as position shift, boundary diffusion, and brightness variation in some sub-mages of the light-field image [19,20]. Based on this, we propose a method for rectifying the distorted light-field image using raw white images. First, the microlens center is calibrated to determine the center of each sub-image and divide the light-field image **x** into a set of sub-image regions *R*. Next, the geometric distortion (e.g., position shift and boundary diffusion) is removed by the geometric rectification matrix **P**. The parameters in the matrix **P** are estimated from the coordinates of the extracted feature points in each sub-image. Finally, the grayscale distortion (e.g., brightness variation) is decreased with the grayscale rectification matrix **G**. The grayscale factors can be calculated sequentially from the gray average of the geometric-rectified sub-images. Figure 3 illustrates the rectification procedure of the proposed method.

### 3.1. Microlens Center Calibration

As a result of the manufacturing and assembly accuracy of the camera and the lens aberration, the sub-images, corresponding to the microlenses, are shifted to different degrees. For accurate extraction of feature point information and subsequent geometric and grayscale rectification for each image, it is a prerequisite that the microlens center is calibrated to determine the relationship between the pixel and the microlens.

**Figure 4:**
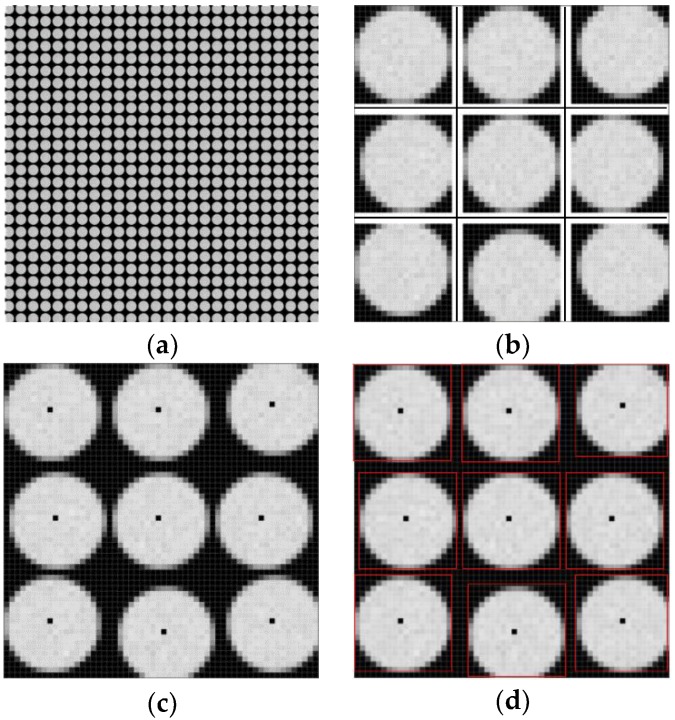
Step-by-step results of microlens center calibration. (**a**) Raw white light-field image captured by plenoptic camera; (**b**) Preliminary division of microlens region; (**c**) Estimated center point; (**d**) Divided sub-image region.

To calibrate all microlens images, a uniform white plate (shown in Figure 1) is adopted to capture a raw white light-field image, which consists of individual microlens sub-images, as shown in Figure 4a. Because of lens vignetting, the gray value maximum in each white sub-image approximates the microlens center theoretically. In order to avoid the effect of inhomogeneous diffuse reflection and image noise on the center calibration, we take statistics to sum the pixel gray values of the raw image by rows and columns:

(2) $$S_{row}\left(x\right)=\sum_{y=1}^{N}I\left(x,y\right),\;x=1,2,\ldots ,M$$

(3) $$S_{col}\left(y\right)=\sum_{x=1}^{M}I\left(x,y\right),\;y=1,2,\ldots ,N$$

where I(x,y) denotes the gray value of the pixel in the *x*-th row and *y*-th column of the image, $S_{row}\left(x\right)$ and $S_{col}\left(y\right)$ denotes the sum of the gray values of the pixels in the *x*-th and the *y*-th column, respectively, and the raw image resolution is *M* (H) × *N* (W).

The thresholds $Th_{row}=1.5\times \underset{1\leq x\leq M}{\min}S_{row}\left(x\right)$ and $Th_{col}=1.5\times \underset{1\leq x\leq N}{\min}S_{col}\left(y\right)$ are set to screen the above results. The row, $S_{row}\left(x\right)$ of which is below the threshold $Th_{row}$, is selected as the horizontal boundary of the sub-image, and the column with $S_{col}\left(y\right)$ below the threshold $Th_{col}$ is selected as the vertical boundary. Through the four adjacent boundaries, the raw image is preliminarily divided into a number of microlens regions $R^{\prime }:R_{1,1}^{\prime },R_{1,2}^{\prime },\ldots ,R_{m,n}^{\prime },\ldots$, where the subscripts *m*,*n* represent the *m*-th row and *n*-th column divided region, as shown in Figure 4b.

After acquiring all the microlens regions, the sub-image center can be located based on the division result. First, we calculate the sum of the pixel gray values in each row and each column of the region $R_{m,n}^{\prime }$:

(4) $$S_{row}^{m,n}\left(x\right)=\sum_{y=c}^{d}I\left(x,y\right),\;x=a,\cdots ,b$$

(5) $$S_{col}^{m,n}\left(y\right)=\sum_{x=a}^{b}I\left(x,y\right),\;y=c,\ldots ,d$$

where *a* and *b* denote the horizontal boundaries of the region $R_{m,n}^{\prime }$, and *c* and *d* denote the vertical boundaries. Then, the pixel point whose the row and column with the largest sum of gray values in the region $R_{m,n}^{\prime }$ is determined as the sub-image center $c_{m,n}$, that is:

(6) $$c_{m,n}\left(x_{c},y_{c}\right)=\left(\arg\underset{a\leq x\leq b}{\max}\left\{S_{row}^{m,n}\left(x\right)\right\},\arg\underset{c\leq y\leq d}{\max}\left\{S_{col}^{m,n}\left(y\right)\right\}\right)$$

Figure 4c shows the center calibration results of microlens sub-images. Finally, according to the center coordinates and the number of pixels *l* × *l* covered by sub-image, the raw image is divided into the sub-image regions $R:R_{1,1},R_{1,2},\ldots ,R_{m,n},\ldots ,R_{N_{H},H_{W}}$, which correspond to *N_W_* × *N_H_* microlenses, respectively, as shown in Figure 4d.

The calibration method proposed in this paper takes into account the sub-image shift caused by the MLA errors. On the basis of the preliminary division of the microlens region, the center points are further obtained by summing the rows and columns of pixel gray values in each region, so that accurate center location and region division for all sub-images can be achieved. As shown in Figure 4c,d, when there are local offsets in the raw white light-field image, the proposed method can still calibrate the microlens center and divide the corresponding sub-image regions, which provides the basis for the geometric and grayscale rectification.

### 3.2. Geometric Rectification

From the imaging principle of the plenoptic camera and the simulation results of MLA errors [19,20], it is known that the geometric distortion of the light-field image typically manifests as the sub-image translation, rotation and scaling, or the superposition of the arbitrary aforementioned forms. Therefore, we use a geometric error matrix and feature point to establish the correspondence between the error sub-image and the ideal sub-image. Consider a feature point $P_{0}\left(x_{m,n},y_{m,n}\right)$ in the error sub-image region $R_{m,n}$, and its coordinates are $\left(u_{m,n},v_{m,n}\right)$ in the ideal sub-image. For a 2D plane, the coordinates of *P*_0_ between these two sub-images satisfy:

(7) $$\left[\begin{array}{c} x_{m,n}\\ y_{m,n} \end{array}\right]=R_{m,n}\left[\begin{array}{c} u_{m,n}\\ v_{m,n} \end{array}\right]+T_{m,n}$$

where $R_{m,n}=\left[\begin{array}{cc} r_{m,n}^{11} & r_{m,n}^{12}\\ r_{m,n}^{21} & r_{m,n}^{22} \end{array}\right]$ represents the rotation and scaling of the sub-image region $R_{m,n}$, and $T_{m,n}={\left[t_{m,n}^{13},t_{m,n}^{23}\right]}^{T}$ represents the translation of the sub-image region $R_{m,n}$. Equation (7) can be written in homogeneous coordinates as:

(8) $$\left[\begin{array}{c} x_{m,n}\\ y_{{}_{m,n}}\\ 1 \end{array}\right]=\left[\begin{array}{ccc} r_{m,n}^{11} & r_{m,n}^{12} & t_{m,n}^{13}\\ r_{im,n}^{21} & r_{m,n}^{22} & t_{m,n}^{23}\\ 0 & 0 & 1 \end{array}\right]\left[\begin{array}{c} u_{m,n}\\ v_{m,n}\\ 1 \end{array}\right]=P_{m,n}^{\prime }\left[\begin{array}{c} u_{m,n}\\ v_{m,n}\\ 1 \end{array}\right]$$

where $P_{m,n}^{\prime }$ is the geometric error matrix of the sub-image region $R_{m,n}$.

**Figure 5:**
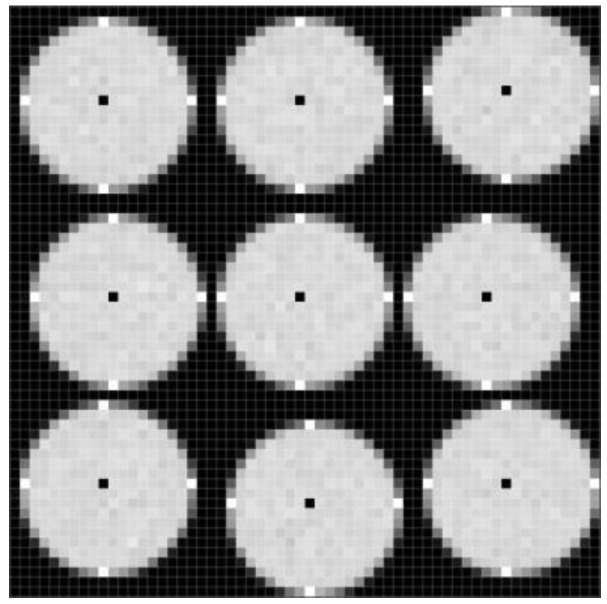
Extracted sub-image feature points for solving geometric error matrix.

According to Equation (8), $P_{m,n}^{\prime }$ can be estimated when only three feature points are set theoretically. To improve calculation accuracy without compromising data processing speed, we adopt five feature points to solve the geometric error matrix of each sub-image. As illustrated in Figure 5, the feature point at the center is the calibrated center point of the sub-image in Section 3.1, and the feature points at the edges are the four edge points in the central row and column of the sub-image.

For the extraction of edge feature points, we use the Sobel template [37,38] to convolve with the pixels (x,y) in the central row and column (i.e., the *x_c_*-th row and *y_c_*-th column of the sub-image with center $c_{m,n}$) of the sub-image region $R_{m,n}$:

(9) $$\begin{array}{l} G_{X}\left(x,y\right)=|I\left(x-1,y+1\right)+2I\left(x,y+1\right)+I\left(x+1,y+1\right)\\ \;-\left[I\left(x-1,y-1\right)+2I\left(x,y-1\right)+I\left(x+1,y-1\right)\right]| \end{array}$$

(10) $$\begin{array}{l} G_{Y}\left(x,y\right)=|I\left(x+1,y-1\right)+2I\left(x+1,y\right)+I\left(x+1,y+1\right)\\ \;-\left[I\left(x-1,y-1\right)+2I\left(x-1,y\right)+I\left(x-1,y+1\right)\right]| \end{array}$$

where $G_{X}\left(x,y\right)$ and $G_{Y}\left(x,y\right)$ are the horizontal and vertical convolution results, respectively. The gradient of the pixel point (x,y) is given by:

(11) $$\nabla \left[I\left(x,y\right)\right]=\sqrt{G_{X}{\left(x,y\right)}^{2}+G_{Y}{\left(x,y\right)}^{2}}$$

The pixel points with maximum gradient value in the directions of upper, lower, left, and right are extracted as the edge feature points, labelled as $p_{m,n}^{N}$, $p_{m,n}^{S}$, $p_{m,n}^{W}$, and $p_{m,n}^{E}$, with the coordinates $p_{m,n}^{N}\left(x_{1},y_{c}\right)$, $p_{m,n}^{S}\left(x_{2},y_{c}\right)$, $p_{m,n}^{W}\left(x_{c},y_{1}\right)$ and $p_{m,n}^{E}\left(x_{c},y_{2}\right)$.

When $P_{m,n}^{\prime }$ is estimated, for arbitrary pixel point $P^{\prime }\left(x_{m,n}^{\prime },y_{m,n}^{\prime }\right)$ in the error sub-image region $R_{m,n}$, the coordinates corresponding to the ideal sub-image are $\left(u_{m,n}^{\prime },v_{m,n}^{\prime }\right)$ via a coordinate transformation. Thus, the correspondence between the error coordinates and the ideal coordinates of each pixel in the sub-image can be deduced with the inverse matrix $P_{m,n}={\left(P_{m,n}^{\prime }\right)}^{-1}$, thereby rectifying the sub-image geometric distortion, that is:

(12) $$\left[\begin{array}{c} u_{m,n}^{\prime }\\ v_{m,n}^{\prime }\\ 1 \end{array}\right]=P_{m,n}\left[\begin{array}{c} x_{m,n}^{\prime }\\ y_{m,n}^{\prime }\\ 1 \end{array}\right]={\left[\begin{array}{ccc} r_{m,n}^{11} & r_{m,n}^{12} & t_{m,n}^{13}\\ r_{m,n}^{21} & r_{m,n}^{22} & t_{m,n}^{23}\\ 0 & 0 & 1 \end{array}\right]}^{-1}\left[\begin{array}{c} x_{m,n}^{\prime }\\ y_{m,n}^{\prime }\\ 1 \end{array}\right]$$

where $P_{m,n}$ is the geometric rectification matrix of the sub-image region $R_{m,n}$.

The coordinates of the feature points in each error sub-image and its ideal sub-image are extracted from raw white light-field images. Using the extracted coordinates, the error matrix parameters and the corresponding rectification matrix of each sub-image are determined by Equations (8) and (12), and finally **P**, the geometric rectification matrix of the light-field image can be composed as follows:

(13) $$P=\left[\begin{array}{cccc} P_{1,1} & P_{1,2} & \cdots & P_{1,n}\\ P_{2,1} & P_{2,2} & \cdots & P_{2,n}\\ \vdots & \vdots & \ddots & \vdots \\ P_{m,1} & P_{m,2} & \cdots & P_{i,n} \end{array}\right]$$

For the light-field image **x** to be rectified, with the geometric rectification matrix **P**, the coordinates of all the pixels in the light-field image are transformed according to Equation (14), and we obtain the geometric-rectified image $x^{\prime }$.

(14) $$x^{\prime }=Px$$

### 3.3. Grayscale Rectification

The final step of the proposed method is to rectify the grayscale distortion in light-field image by a grayscale rectification matrix **G**. Since some surface errors of the MLA alter the luminance and contrast of the sub-image, the brightness of light-field image is non-uniform, which affects the 3D reconstruction results. Therefore, grayscale rectification is considered to recover the brightness uniformity of the light-field image after geometric distortion rectification.

For a geometric-rectified sub-image region $R_{m,n}$, the grayscale factor $g_{m,n}$ is defined as:

(15) $$g_{m,n}=\frac{\mu_{S_{m,n}}}{\mu_{R_{m,n}}}$$

where $\mu_{R_{m,n}}$ and $\mu_{S_{m,n}}$ are the gray average of the sub-image region $R_{m,n}$ and the corresponding ideal sub-image image, respectively. $\mu_{R_{m,n}}$ and $\mu_{S_{m,n}}$ can be calculated from:

(16) $$\mu_{R_{m,n}}=\frac{1}{l^{2}}\sum_{x=1}^{l}\sum_{y=1}^{l}I_{R_{m,n}}\left(x,y\right)$$

(17) $$\mu_{S_{m,n}}=\frac{1}{l^{2}}\sum_{x=1}^{l}\sum_{y=1}^{l}I_{S_{m,n}}\left(x,y\right)$$

where $I_{R_{m,n}}\left(x,y\right)$ and $I_{R_{m,n}}\left(x,y\right)$ denote the gray values of the pixel point (x,y) in the two images, respectively, and $l^{2}$ is the number of the pixels covered by the sub-image.

The grayscale factor of each sub-image in the raw image is calculated sequentially from Equation (15), obtaining the light-field image grayscale rectification matrix:

(18) $$G=\left[\begin{array}{cccc} g_{1,1} & g_{1,2} & \cdots & g_{1,n}\\ g_{2,1} & g_{2,2} & \cdots & g_{2,n}\\ \vdots & \vdots & \ddots & \vdots \\ g_{m,1} & g_{m,2} & \cdots & g_{m,n} \end{array}\right]$$

For the geometric-rectified light-field image $x^{\prime }$, the brightness deviation is modified by multiplying the gray value of all the pixels in each sub-image with the corresponding grayscale factor to acquire the grayscale-rectified image $x^{\prime \prime }$, that is:

(19) $$x^{\prime \prime }=Gx^{\prime }$$

In the proposed method, including center calibration, geometric rectification and grayscale rectification, only the raw white light-field image (error image) captured by a plenoptic camera is used. Moreover, the white image simulated from the plenoptic imaging system with the standard parameters under the same conditions is used as an ideal image to jointly solve the geometric rectification matrix **P** and grayscale rectification matrix **G** of the light-field image. Once the parameters of the two matrices are determined, they can be applied to the targeted geometric and grayscale rectification for other images of any scene captured by the same plenoptic camera.

## 4. Results and Analysis

**Figure 6:**
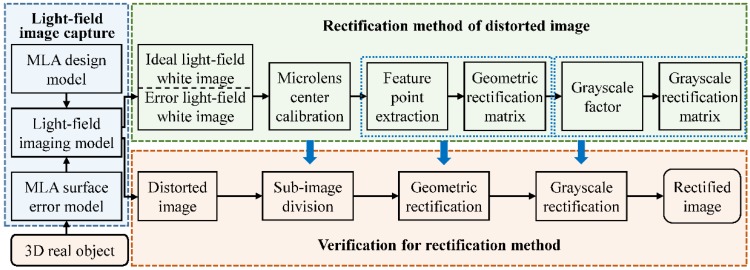
Flowchart of our simulation experiment.

In this section, we investigate the rectification accuracy and applicable error range of the proposed method for the three basic errors of the MLA, and verify the rectification method for light-field images of real objects. Figure 6 shows the flowchart of the simulation experiments. First, the ideal MLA model and the surface error model with different types and magnitudes are added to the light-field imaging model established in Section 2.1, to obtain the ideal white image and the error white image. Second, the complete parameters of the geometric and grayscale rectification matrix are obtained by the method proposed in Section 3, and the rectified white image is used to quantitatively analyze the rectification effect. Third, the real objects are imaged by the light-field imaging model with MLA errors, and the light-field images before and after rectification, sub-aperture images and refocused images, respectively, are evaluated to verify the effectiveness and reliability of the proposed method.

**Figure 7:**
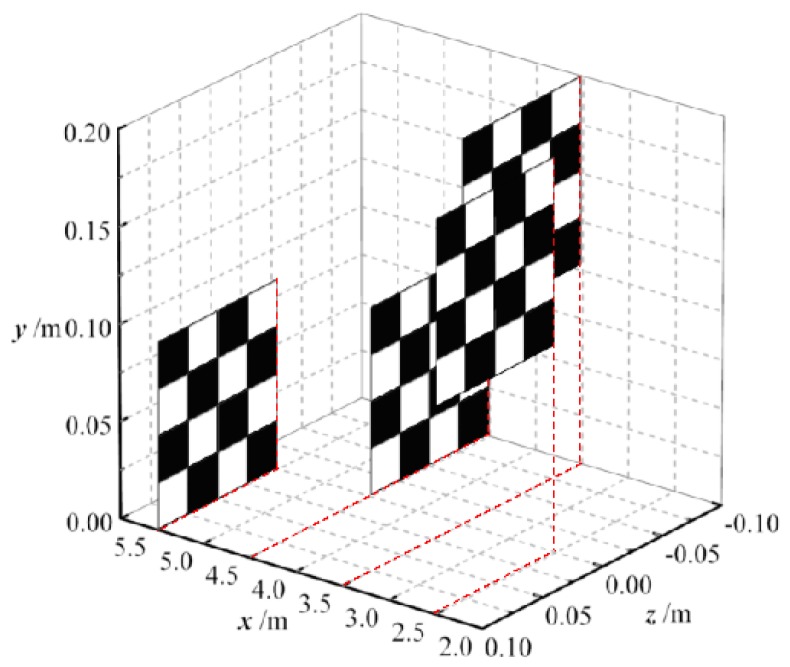
Layout of real objects in light-field imaging.

The real objects adopted in this paper are a set of square plates at different depths as shown in Figure 7. Each plate size is 10 × 10 cm, and consists of black and white squares with a side length of 2.5 cm. They are placed 2.5, 3.5, 4.5 and 5.5 m away from the main lens plane. We used Monte Carlo algorithm for the simulation on a 2.40 GHz Intel^®^ Xeon^®^ E5-2680v4 server with 128.0 GB of RAM. To guarantee an adequate number of rays and improve computational efficiency, parallel computation with 10 threads was performed. The total number of rays in the simulation was 3 × 10^10^.

### 4.1. Pitch Error

**Figure 8:**
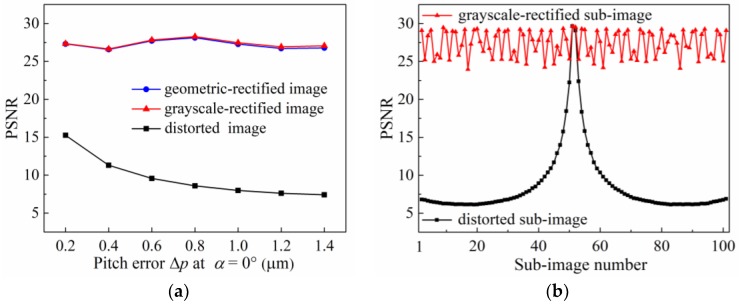
PSNR results for pitch error in light-field image. (**a**) Overall PSNR of raw white light-field images under different pitch error conditions; (**b**) Local PSNR of each sub-image on the diagonal of the raw image with a pitch error Δ*p* = 1.4 μm at *α* = 90°.

When a pitch error exists in the MLA of a plenoptic camera, the center position of the microlens changes, which causes its optical axis to move, resulting in discontinuity or aliasing between the sub-images formed on the image sensor after rays pass through the MLA. To analyze the rectification accuracy of the proposed method for the distorted light-field images caused by the pitch error, according to typical processing effects in optical freeform surfaces, we add a vertical pitch error (*α* = 90°) in the range 0.2–1.4 μm at intervals of 0.2 μm to the MLA model for the simulation experiments, obtaining distorted white light-field images. Next, using the method proposed in Section 3, geometric rectification and grayscale rectification are sequentially performed on the distorted images. We measure the peak signal to noise ratio (PSNR) of the distorted, geometric-rectified, and grayscale-rectified images, and the results are presented in Figure 8a. The pitch error can cause severe degradation of the light-field image, and the PSNR value of the image decreases as the error increases. The PSNR value of the geometric-rectified image ranges from 26.57 dB to 28.11 dB, and the PSNR value of the grayscale-rectified image ranges from 26.64 dB to 28.27 dB. It indicates that the method can effectively recover image quality from the pitch error distortion.

For further study of the rectification effect on sub-images at different positions in the light-field image, considering the pitch error Δ*p* = 1.4 μm at *α* = 90° as an example, the PSNR results of the sub-images on the diagonal of the distorted image before and after rectification are selected to make plots of the relationship of the sub-image position and the PSNR value. As shown in Figure 8b, for unrectified light-field images, the PSNR value of the sub-image decreases rapidly with the distance from its position to the image center. For grayscale-rectified light field images, the overall quality of the sub-images is significantly improved. The differences in sub-image quality at different positions are small, and the fluctuation range of PSNR values is 24.08–29.63 dB with an average of 27.34 dB.

The fluctuation of sub-image quality after rectification can be owing to the limitation of the pixel size on the image sensor. Because the integer pixel unit is not sufficient to exactly represent the center points of the sub-images, only when the sub-image center coincided with the center of the pixel unit, the geometric distortion of the sub-image can be fully rectified. In other cases, the rectification has a different degree of deviation. As a result, the PSNR values of the rectified sub-images fluctuate within a certain range. As per the above analysis, it is believed that the proposed method can effectively rectify the distorted light-field images in the pitch error range Δ*p* ≤ 1.4 μm. The overall rectification accuracy is greater than 26.64 dB, and the local rectification accuracy is greater than 24.08 dB.

**Figure 9:**
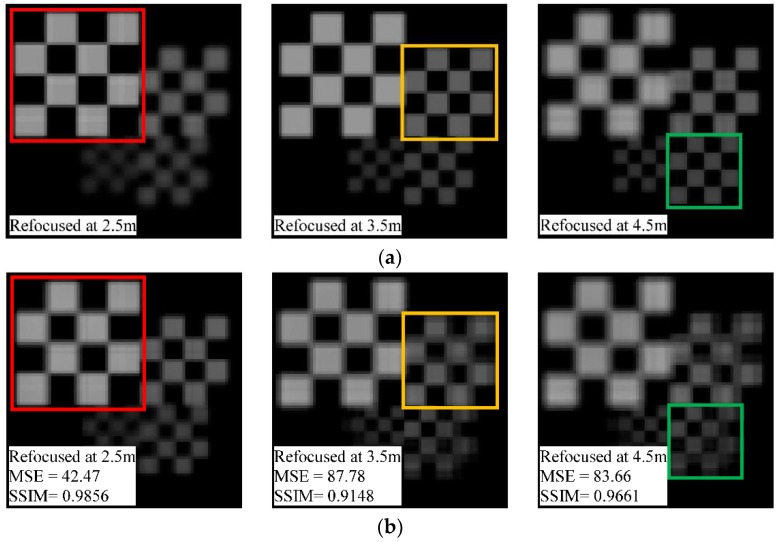

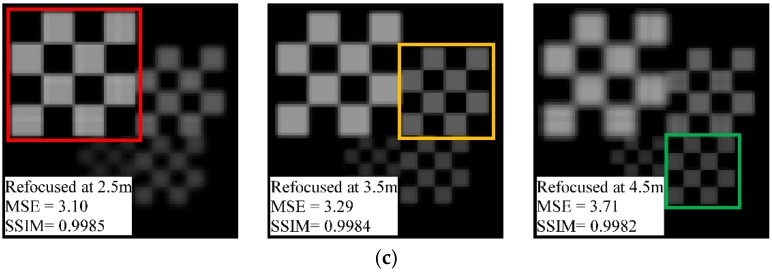
Comparison with refocused images of real object (**a**) from the standard light-field image; (**b**) from the distorted image with a pitch error Δ*p* = 1.4 μm at *α* = 0° and *α*= 90°; and (**c**) from the image rectified by the proposed method. The MSE and SSIM quality evaluation results for each refocused image are marked in its lower left corner.

In order to evaluate the proposed method for real object images, we set a pitch error of Δ*p* = 1.4 μm at *α* = 0° and *α* = 90° in the simulation imaging model for the objects shown in Figure 7, and performed digital refocusing on the raw light-field images before and after rectification. The resultant images that refocus at different positions are shown in Figure 9b,c. For comparison, Figure 9a also shows the refocusing results of the ideal image under standard conditions. It can be seen from Figure 9b that the refocused images computed from the distorted raw image has blurring, aliasing, and stretching deformations to varying degrees, and the distortion becomes more obvious with the refocusing depth, so that an accurate refocused image cannot be obtained at these longer refocusing positions. However, there is slight distortion in the refocused images (Figure 9c) using the rectified raw image, where the object information at different positions is clearly reconstructed. In addition, we also introduce the mean square error (MSE) and structural similarity index (SSIM) [39] to quantitatively characterize the refocused image quality before and after rectification.

The quality evaluation results for each refocused image are marked in its lower left corner. With rectification processing, the grayscale and structural similarity distortions of the refocused images are greatly reduced, and can be approximated as standard refocused images. This indicates that the proposed method can compensate for the pitch error of the MLA and transform the raw light-field image to fulfil the object refocusing requirements.

### 4.2. Radius-of-Curvature Error

**Figure 10:**
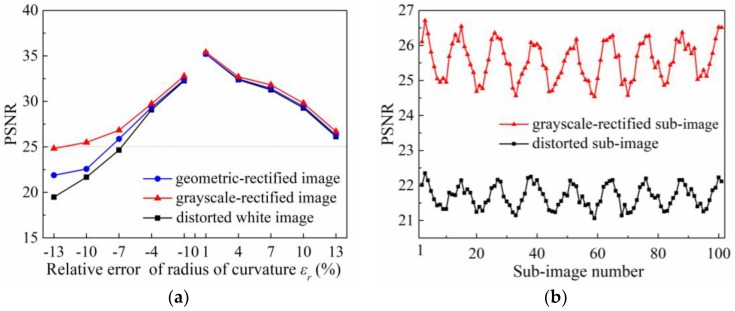
PSNR results for radius-of-curvature error in light-field image. (**a**) Overall PSNR of raw white light-field images under different radius-of-curvature error conditions; (**b**) Local PSNR of each sub-image on the diagonal of the raw image with a relative error *ε_r_* = −10%.

**Figure 11:**
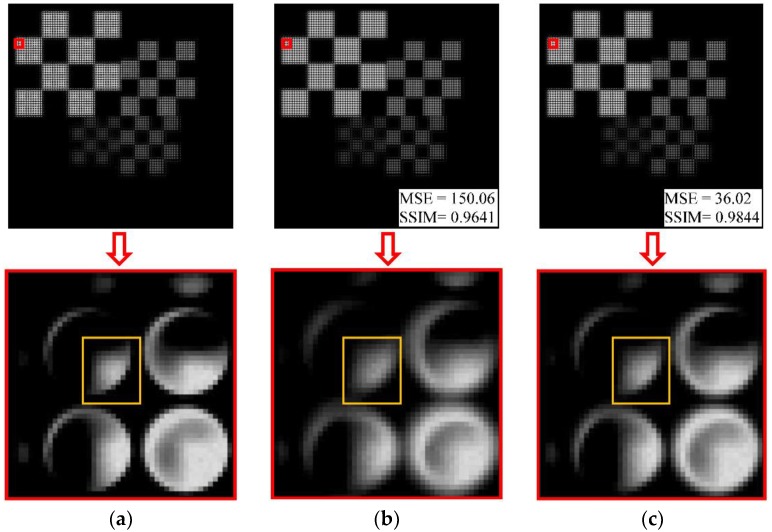
Comparison with partially enlarged light-field images of real object (**a**) under standard conditions; (**b**) with a relative error *ε_r_* = −10%; and (**c**) after geometric rectification. The MSE and SSIM quality evaluation results for each image are marked in its lower right corner.

Radius-of-curvature error changes the focal length of the microlens in the MLA, so that the distance from the erroneous microlens to the sensor does not satisfy the conjugate distance, resulting in a reduced resolution of the raw image captured by the plenoptic camera. To analyze the rectification effect on the light-field image with radius-of-curvature error, we define the relative error of the radius-of-curvature *ε_r_* = Δ*r*/*r*, and set the relative error range as −13% ≤ *ε_r_* ≤ 13%. Figure 10a shows the PSNR results of the distorted raw white light-field image and the corresponding geometric- and grayscale-rectified images under different error conditions. From the plot without rectification, the larger the magnitude of the radius-of-curvature error, the smaller the PSNR value of the raw image before rectification, and negative errors have a more significant effect on the image than positive errors. But in general, the image degradation caused by this error is small. The minimum PSNR within the error range is 19.49 dB. From the plot with rectification, the PSNR value of the raw image is improved, but it still decreases as the magnitude of the error increases. When the relative error *ε_r_* = −10%, the obtained PSNR value of the grayscale-rectified image can be over 25 dB. The degradation degree of the image is low, and the distortion is invisible to the human eye (see partially enlarged standard image and rectified image in the following Figure 11), which indicates that this rectification method is applicable for distorted images that have a relative error $\left|\varepsilon_{r}\right|\leq 10\%$.

In order to explore the local rectification accuracy for the radius-of-curvature error, we calculate the PSNR values of the sub-images on the diagonal of the distorted image with the relative error *ε_r_* = −10%, and the corresponding grayscale-rectified image. As can be seen from the results shown in Figure 10b, the sub-image quality of the raw distorted image varies periodically with the position of the sub-image. After rectification, each sub-image has higher quality, and the average PSNR can reach 25.65 dB. The overall quality of the rectified image still has periodic variations in the range 24.54–26.71 dB, but the period remains unchanged. Moreover, the PSNR values of the sub-images near the center of the image are lower than those of the sub-images at the edge of the image, which becomes more significant after rectification.

Since the error does not change the center position of the microlens sub-image, the periodic variation can be attributed to the slight deviation between the sub-image and its ideal position due to the main lens aberration. The position deviation of the sub-image increases with the distance from the microlens to the MLA center. The sub-images near the center have the small deviations, such that the geometric rectification effect is not obvious. And for the sub-images near the edge, the deviations become larger, and these can be well-rectified using the proposed method. Consequently, the quality of the sub-images near the edge is higher after rectification. From the above analysis, it is confirmed that the proposed method can achieve ideal rectification for the distorted light-field images in the radius-of-curvature relative error range −10% ≤ *ε_r_* ≤ 10%. The overall rectification accuracy is greater than 24.59 dB, and the local rectification accuracy is greater than 24.54 dB.

Figure 11a,b shows the simulated light-field imaging results for the real objects in Figure 7 under standard conditions and with a relative error *ε_r_* = −10% for the MLA. It can be seen from the partially enlarged images that the boundaries of the spots in the distorted image diffuse outwards, crosstalk occurs between adjacent spots, and the definition of the edges of the object pattern (highlighted with a yellow outline) decreases. Using our method to rectify the image in Figure 11b, the rectified light-field image is obtained, as shown in Figure 11c. The edge diffusion and crosstalk of the spots are reduced, and the blurring of the pattern edge is also alleviated. Although there are some distortions in the spot edges, the definition and contrast of the entire image is significantly improved compared to the image without rectification. The MSE value of the image is reduced by 76.00%, and the SSIM value is increased by 2.11%. This result shows that the proposed method can be applied to real object rectification under the radius-of-curvature error condition.

### 4.3. Decenter Error

**Figure 12:**
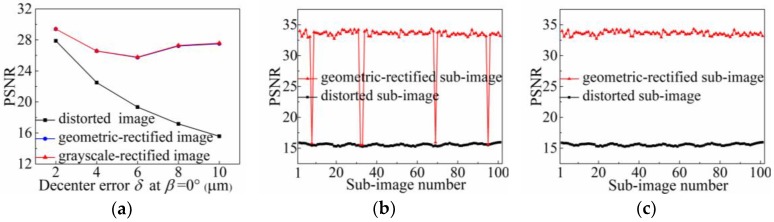
PSNR results for decenter error in light-field image. (**a**) Overall PSNR of raw white light-field images under different decenter error conditions; (**b**) Local PSNR of each sub-image on the diagonal of the raw image with a decenter error *δ* = 10 μm and *β* = 90°; (**c**) Local PSNR results after center modification.

Because of manufacturing technical defects and other uncertainties, the MLA with a double-sided structure may have decentering errors after processing [28,29]. The decenter error causes the optical axes of the microlenses to tilt, resulting in the movement of the sub-images formed on the image sensor. To demonstrate the rectification ability of the proposed method to this error, we set a decenter error ranging from 2 μm to 10 μm, and the angle *β* with the horizontal direction of 90° in the MLA model, for imaging simulation, and sequentially calculated the PSNR values of these raw white images before and after rectification. From the results shown in Figure 12a, the influence of the decenter error on the light-field image is related to the magnitude of the error. As the error increases, the degradation of the raw image gradually is aggravated and the PSNR value also decreases. After rectification, the raw images restore quality with the high PSNR values for all the different decenter errors. The PSNR values of the rectified images are mostly concentrated between 26 dB and 28 dB, and whether or not grayscale-rectified, the PSNR values of the images are basically the same. This indicates that for the image distortion caused by the decenter error, the quality of the light-field image can be recovered only by the geometric rectification method, without performing grayscale rectification.

We select the raw white image with the decenter error of *δ* = 10 μm at *β* = 90° to investigate the accuracy of the geometric rectification method for the sub-images at different positions in the decenter error image. Figure 12b shows the PSNR results of the sub-images on the diagonal of the distorted and grayscale-rectified images. The PSNR values of the unrectified sub-images, show a periodic fluctuation, in the range 15.25–15.95 dB. After geometric rectification, the PSNR values of most sub-images can reach more than 32.73 dB, the quality of which is approximately equal to the corresponding standard sub-images. However, for a few sub-images, the rectification results are not ideal and the PSNR values are still lower than 16 dB, seriously affecting the rectification accuracy of the entire image. The main reason we consider to be responsible for this is that the vignetting of the microlens suffers from the tilt of the optical axis caused by the decenter error. For the sub-images corresponding to the microlenses at certain positions, the center points determined by the center calibration method may have a deviation (not more than one pixel), and the subsequent extraction of edge feature points is also inaccurate, so that these distorted sub-images cannot be recovered with the geometric rectification.

As a verification, we modified the center coordinates of the four sub-images with low PSNR values in Figure 12b and re-performed the rectification processing. From the results shown in Figure 12c, the quality of these four sub-images with the center modification is significantly improved after the geometric rectification, and the average PSNR of the entire set of sub-images is 33.60 dB. Based on the above analysis, we confirm that the proposed geometric rectification method can perfectly rectify distorted light-field images in the decenter error range *δ* ≤ 10 μm. The overall rectification accuracy is greater than 25.88 dB, and the local rectification accuracy with center modification is greater than 32.82 dB.

**Figure 13:**
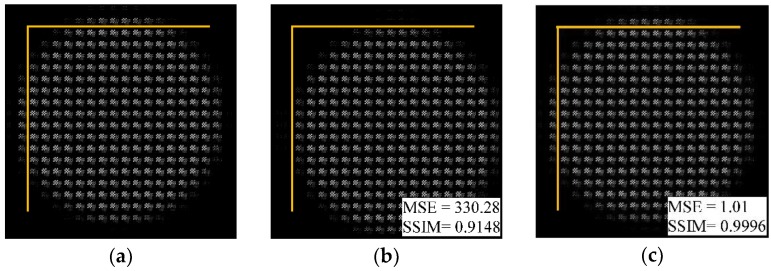
Comparison with sub-aperture images of real object (**a**) from the standard light-field image; (**b**) from the distorted image with a decenter error *δ =* 10 μm at *β* = 0° and *β* = 90°, and (**c**) from the image rectified by the proposed method. The MSE and SSIM quality evaluation results for each sub-aperture image are marked in its lower right corner.

Further, we add a decenter error of 10 μm to the MLA model in the horizontal and vertical two directions for the imaging simulation of the real objects. Figure 13a–c shows the sub-aperture images of the object image under standard conditions, with the decenter error, and after geometric rectification, respectively. As shown in Figure 13a,b, the sub-aperture image with the error is shifted along the horizontal and vertical directions based on the boundary of the standard sub-aperture image (shown by the yellow line), causing image degradation in grayscale and structural similarity, and the dislocation aliasing of the subsequent refocused images. After the rectification of the light-field image, the sub-aperture image has no offset. The MSE and SSIM values of the sub-aperture image are 1.01 and 0.9996, which can be considered to be very similar to the standard sub-aperture image. The effectiveness of the rectification method for real object imaging with the decenter error is verified.

### 4.4. Combined Error

**Figure 14:**
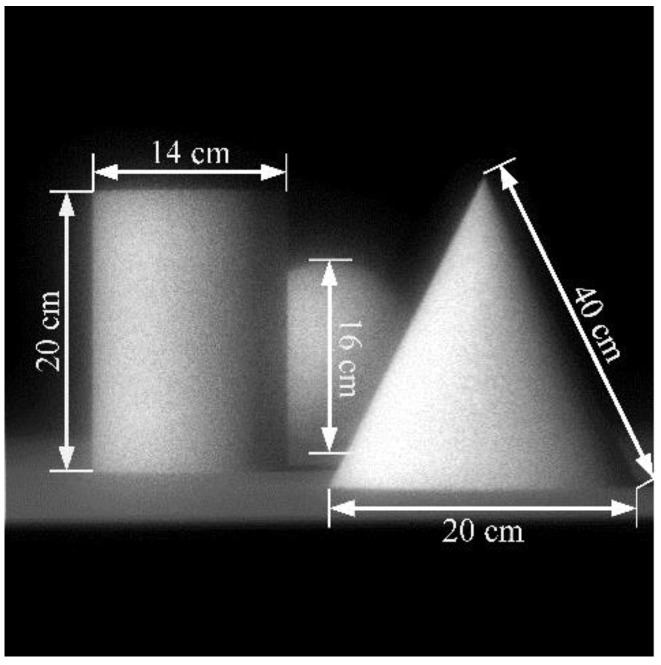
Real 3D objects in performance test for combined errors.

The surface errors of the MLA after processing are complicated such that there may be two or three different errors in one microlens. Based on the analysis of the single error, we further test the performance of the proposed method for the combined errors. Considering a complex scene with multiple objects, the real objects used in this section are a set of 3D geometric entities with occlusion, as shown in Figure 14. The cone, cylinder, and sphere are placed at 4.5, 5.0, and 6.0 m from the main lens plane. The MLA surface error model is set to a multi-error combination condition, in which the pitch error is Δ*p* = 1.4 μm at *α* = 90°, the radius-of-curvature relative error is *ε_r_* = −10% and the decenter error is *δ* = 10 μm at *β* = 0°, for imaging experiments.

**Figure 15:**
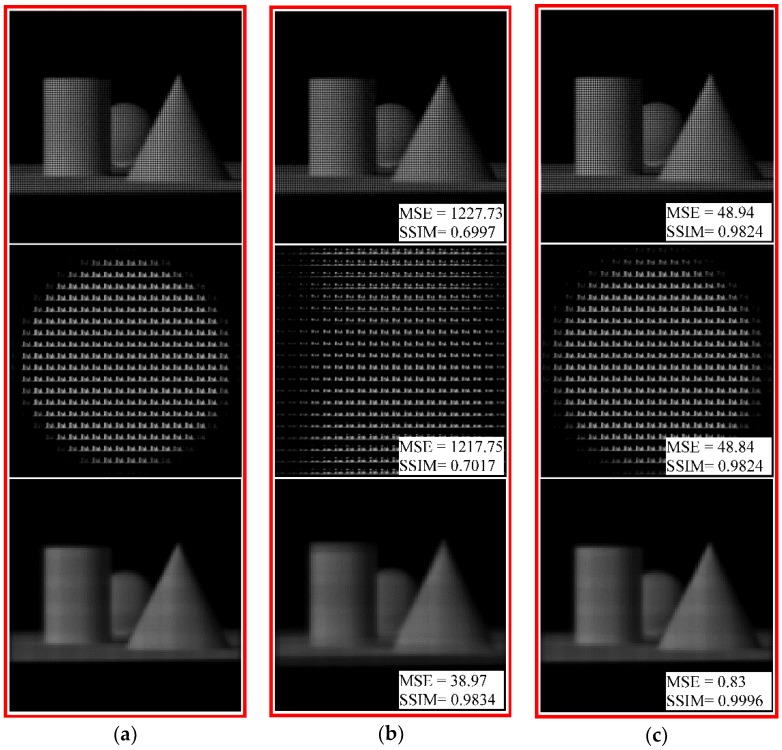
Comparison with light-field images, sub-aperture images and refocused images of real 3D objects (**a**) under standard conditions; (**b**) with a combined error; and (**c**) after grayscale rectification. The MSE and SSIM quality evaluation results for each sub-aperture image are marked in its lower right corner.

Figure 15 shows the light-field images and the corresponding sub-aperture images and refocused images under standard conditions, with the combined error, and after grayscale rectification, respectively. As can be seen from Figure 15b, the distorted images appear as a superposition of the distortion characteristics of each single error, such as aliasing and stretching deformation caused by the pitch error, crosstalk due to the radius-of-curvature error, and image shift from the decenter error. The degradation degree of the overall image is very high. The rectified images, as shown in Figure 15c, have no obvious distortions and deforms, showing clear images of the real 3D objects.

## 5. Disscussion

In this paper, we have proposed a local rectification method for the distorted light-field image caused by MLA surface errors using a raw white image. The method includes calibrating the sub-image center of the microlens, and sequentially rectifying the geometric distortion and grayscale deviation of each sub-image. Based on the basic error model of the MLA and the plenoptic camera imaging model established in this paper, we analyzed the accuracy and applicable error range of the proposed method for rectifying the pitch error, radius-of-curvature error, and decenter error. Further, we also evaluated the light-field image quality of real objects before and after rectification under different error conditions including single error and combined error by utilizing the MSE and SSIM indexes, verifying the effectiveness and reliability of the proposed rectification method.

The analysis results indicate that the pitch error can cause serious degradation of the light-field image, and the refocused image of the real objects displays blurring, aliasing and stretching deformation. In the pitch error range Δ*p* ≤ 1.4 μm, the proposed method can effectively rectify the distorted image, with overall rectification accuracy greater than 26.64 dB, and local rectification accuracy greater than 24.08 dB. The rectified image can accurately reconstruct the object information at different positions. The radius-of-curvature error can cause periodic fluctuations in the light-field image quality, and in the real object image, the adjacent spots have crosstalk, and the definition of the pattern edges decreases. In the radius-of-curvature error range −10% ≤ *ε_r_* ≤ 10%., the overall rectification accuracy is greater than 24.59 dB, and the local rectification accuracy is greater than 24.54 dB. The MSE value of the rectified object image is reduced by 76.00%, and the SSIM value is increased by 2.11%. For the decenter error, the degradation and distortion of the light-field image increases with the error magnitude, and the sub-aperture image of the object is shifted along the error direction. In the decenter error range *δ* ≤ 10 μm, the light-field image quality can be restored with the geometric rectification method alone, to yield an overall rectification accuracy greater than 25.88 dB, and local correction accuracy with center modification greater than 32.82 dB.

## 6. Conclusions

In conclusion, the rectification method proposed in this paper can achieve a good compensation effect for the image distortion caused by different surface errors of the MLA, effectively reducing loss and deviation of light-field information. The rectified light-field image has a high quality and can meet the requirements of subsequent digital processing and target reconstruction. However, the accuracy of the rectification method is largely affected by the calibration result of the sub-image centers. We determined the center points of each microlens to a certain pixel, but due to the size of the pixels on the sensor, the pixel cannot represent the actual center point very precisely, which may lead to positioning deviation of the center point and inaccurate rectification results, as shown in Figure 8b and Figure 12b. Therefore, future work will consider the use of sub-pixel precision for location of the center points to improve the accuracy of this method.

## Figures and Tables

**Table 1:** Main parameters of MLA model.

Parameters	Value
Number of microlenses *N_W_* × *N_H_*	102 × 102
Pitch (Side length) *p*	100 μm
Radius of curvature *r*	469 μm
Thickness at the vertex *t*	10 μm
Focal length *f*	420 μm
Refractive index *n* (λ = 632.8 nm)	1.56

**Table 2:** Mathematical Model of MLA surface error.

Errors	Surface Description Equations
Pitch error Δ*p*	${\left[x\mp \left(r-t/2\right)\right]}^{2}+{\left[y-\left(y_{0}-(m-1/2)p+\Delta p\sin\alpha \right)\right]}^{2}+{\left[z-\left(z_{0}+(n-1/2)p+\Delta p\cos\alpha \right)\right]}^{2}=r^{2}$
Radius-of-curva-ture error Δ*r*	${\left[x\mp \left(r-t/2\right)\right]}^{2}+{\left[y-\left(y_{0}-(m-1/2)p\right)\right]}^{2}+{\left[z-\left(z_{0}+(n-1/2)p\right)\right]}^{2}={\left(r+\Delta r\right)}^{2}$
Decenter error *δ*	${\left[x-\left(r-t/2\right)\right]}^{2}+{\left[y-\left(y_{0}-(m-1/2)p\right)\right]}^{2}+{\left[z-\left(z_{0}+(n-1/2)p\right)\right]}^{2}=r^{2}$ ${\left[x+\left(r-t/2\right)\right]}^{2}+{\left[y-\left(y_{0}-(m-1/2)p+\delta \sin\beta \right)\right]}^{2}+{\left[z-\left(z_{0}+(n-1/2)p+\delta \cos\beta \right)\right]}^{2}=r^{2}$

## References

[1] Levoy M. (2006). Light fields and computational imaging. Computer.

[2] Ng R., Levoy M., Brédif M., Duval G., Horowitz M., Hanrahan P. (2005). Light field photography with a hand-held plenoptic camera. Comput. Sci. Tech. Rep..

[3] Levoy M., Ng R., Adams A., Footer M., Horowitz M. (2006). Light field microscopy. ACM Trans. Graph..

[4] Georgiev T., Lumsdaine A. (2010). Focused plenoptic camera and rendering. J. Electron. Imaging.

[5] Antensteiner D., Štolc S., Pock T. (2018). A review of depth and normal fusion algorithms. Sensors.

[6] Rodríguez M., Magdaleno E., Pérez F., García C. (2017). Automated software acceleration in programmable logic for an efficient NFFT algorithm implementation: A case study. Sensors.

[7] Pérez J., Magdaleno E., Pérez F., Rodríguez M., Hernández D., Corrales J. (2014). Super-Resolution in plenoptic cameras using FPGAs. Sensors.

[8] Sun J., Xu C., Zhang B., Hossain M.M., Wang S., Qi H., Tan H. (2016). Three-dimensional temperature field measurement of flame using a single light field camera. Opt. Express.

[9] Yuan Y., Liu B., Li S., Tan H. (2016). Light-field-camera imaging simulation of participatory media using Monte Carlo method. Int. J. Heat Mass Transf..

[10] Kim S., Ban Y., Lee S. (2014). Face liveness detection using a light field camera. Sensors.

[11] Fahringer T.W., Lynch K.P., Thurow B.S. (2015). Volumetric particle image velocimetry with a single plenoptic camera. Meas. Sci. Technol..

[12] Chen H., Sick V. (2017). Three-dimensional three-component air flow visualization in a steady-state engine flow bench using a plenoptic camera. SAE Int. J. Engines.

[13] Skinner K.A., Johnson-Roberson M. Towards real-time underwater 3D reconstruction with plenoptic cameras. Proceedings of the 2016 IEEE/RSJ International Conference on in Intelligent Robots and Systems (IROS).

[14] Dong F., Ieng S.H., Savatier X., Etienne-Cummings R., Benosman R. (2013). Plenoptic cameras in real-time robotics. Int. J. Robot. Res..

[15] Liu X., Zhang X., Fang F., Zeng Z., Gao H., Hu X. (2015). Influence of machining errors on form errors of microlens arrays in ultra-precision turning. Int. J. Mach. Tools Manuf..

[16] Cao A., Pang H., Wang J., Zhang M., Chen J., Shi L., Deng Q., Hu S. (2017). The Effects of Profile Errors of Microlens Surfaces on Laser Beam Homogenization. Micromachines.

[17] Thomason C.M., Fahringer T.F., Thurow B.S. Calibration of a microlens array for a plenoptic camera. Proceedings of the 52nd AIAA Aerospace Sciences Meeting.

[18] Li S., Yuan Y., Zhang H., Liu B., Tan H. (2016). Microlens assembly error analysis for light field camera based on Monte Carlo method. Opt. Commun..

[19] Li S., Yuan Y., Liu B., Wang F., Tan H. (2018). Influence of microlens array manufacturing errors on light-field imaging. Opt. Commun..

[20] Li S., Yuan Y., Liu B., Wang F., Tan H. (2018). Local error and its identification for microlens array in plenoptic camera. Opt. Lasers Eng..

[21] Shi S., Wang J., Ding J., Zhao Z., New T.H. (2016). Parametric study on light field volumetric particle image velocimetry. Flow Meas. Instrum..

[22] Fahringer T., Thurow B. The effect of grid resolution on the accuracy of tomographic reconstruction using a plenoptic camera. Proceedings of the 51st AIAA Aerospace Sciences Meeting Including the New Horizons Forum and Aerospace Exposition.

[23] Kong X., Chen Q., Wang J., Gu G., Wang P., Qian W., Ren K., Miao X. (2018). Inclinometer assembly error calibration and horizontal image correction in photoelectric measurement systems. Sensors.

[24] Lourenço M., Barreto J.P., Francisco V. (2012). sRD-SIFT: Keypoint detection and matching in images with radial distortion. IEEE Trans Robot..

[25] Furnari A., Farinella G.M., Bruna A.R., Battiato S. (2017). Affine covariant features for fisheye distortion local modeling. IEEE Trans. Image Process..

[26] Cruz-Mota J., Bogdanova I., Paquier B., Bierlaire M., Thiran J.P. (2012). Scale invariant feature transform on the sphere: Theory and applications. Int. J. Comput. Vis..

[27] Jin J., Cao Y., Cai W., Zheng W., Zhou P. An effective rectification method for lenselet-based plenoptic cameras. Proceedings of the SPIE/COS Photonics Asia on Optoelectronic Imaging and Multimedia Technology IV.

[28] Dansereau D.G., Pizarro O., Williams S.B. Decoding, calibration and rectification for lenselet-based plenoptic cameras. Proceedings of the 2013 IEEE Conference on Computer Vision and Pattern Recognition (CVPR).

[29] Cho D., Lee M., Kim S., Tai Y.W. Modeling the calibration pipeline of the Lytro camera for high quality light-field image reconstruction. Proceedings of the 2013 IEEE International Conference on Computer Vision (ICCV).

[30] Li T., Li S., Li S., Yuan Y., Tan H. (2016). Correction model for microlens array assembly error in light field camera. Opt. Express.

[31] Mukaida M., Yan J. (2017). Ductile machining of single-crystal silicon for microlens arrays by ultraprecision diamond turning using a slow tool servo. Int. J. Mach. Tools Manuf..

[32] Liu B., Yuan Y., Li S., Shuai Y., Tan H. (2015). Simulation of light-field camera imaging based on ray splitting Monte Carlo method. Opt. Commun..

[33] Shih Y.M., Kao C.C., Ke K.C., Yang S.Y. (2017). Imprinting of double-sided microstructures with rapid induction heating and gas-assisted pressuring. J. Micromech. Microeng..

[34] Zhao Z., Hui M., Liu M., Dong L., Liu X., Zhao Y. (2015). Centroid shift analysis of microlens array detector in interference imaging system. Opt. Commun..

[35] Huang C.Y., Hsiao W.T., Huang K.C., Chang K.S., Chou H.Y., Chou C.P. (2011). Fabrication of a double-sided micro-lens array by a glass molding technique. J. Micromech. Microeng..

[36] Xie D., Chang X., Shu X., Wang Y., Ding H., Liu Y. (2015). Rapid fabrication of thermoplastic polymer refractive microlens array using contactless hot embossing technology. Opt. Express.

[37] Furnari A., Farinella G.M., Bruna A.R., Battiato S. Generalized Sobel filters for gradient estimation of distorted images. Proceedings of the 2015 IEEE Conference on Image Processing (ICIP).

[38] Furnari A., Farinella G.M., Bruna A.R., Battiato S. (2017). Distortion adaptive Sobel filters for the gradient estimation of wide angle images. J. Vis. Commun. Image Represent..

[39] Wang Z., Bovik A.C., Sheikh H.R., Simoncelli E.P. (2004). Image quality assessment: From error visibility to structural similarity. IEEE Trans. Image Process..

